# Editorial: Hallucinations from the perspective of altered experiences of self: A multidisciplinary approach

**DOI:** 10.3389/fpsyt.2022.1040969

**Published:** 2022-10-11

**Authors:** Massoud Stephane, Clara S. Humpston, Cherise Rosen, Aaron L. Mishara

**Affiliations:** ^1^Department of Psychiatry, Oregon Health and Science University, Portland, OR, United States; ^2^Department of Psychology, University of York, York, United Kingdom; ^3^Department of Psychiatry, University of Illinois at Chicago, Chicago, IL, United States; ^4^California Institute of Integral Studies, San Francisco, CA, United States

**Keywords:** hallucinations, self experience, psychosis, inner speech and thought, agency

Altered experiences of the self have been often described in psychosis (1) and reported in relation to hallucinations in particular those in the verbal modality (2). With respect to the latter, decades long research has examined one aspect of self experiences, that of the agency of inner speech; and has demonstrated abnormality considered to lead hallucinators to experience their own verbal thoughts as those of others (3). However, altered agency by itself fails short of explaining the complex phenomenology of hallucinations (4). For example, verbal hallucinations are often described as voices (sounds), not thoughts, and are not always experienced as the voices of others. Furthermore, the experiences of the self are no less complex and involves more than agency.

Since the paradigm shifting work of Descartes (5), the study of consciousness and related sense of self has been extensive. Although understanding the experience of self remains controversial, recent research point to a model, originally formulated by James (6), consisting of multiple domains of experience, including: minimal self, narrative self, embodied self, agency and ownership (7, 8).

In line with the above, previous hallucinations research pointed to altered experiences in multiple self domains. In addition to the above-mentioned agency externalization, spatial externalization of inner verbal thoughts was reported (9), and was found independent from agency externalization at phenomenological and cognitive levels (2, 10). Recent research also points another altered experience of self-that of the loudness of the inner voice (11). It is noteworthy that in a more recent research (12), López-Silva et al. have shown that both thought insertion (an external agent inserting thoughts) and thought broadcasting (thoughts leaving the bodily boundary-head) are associated with hallucinations. This research, consistent with the previously noted similarity between verbal hallucinations and thought insertion (13), suggests that un underlying abnormalities in two domains of self experience (agency and internal space) would result in verbal hallucinations, thought insertion or thought broadcasting or combinations of these depending on other factors. Currently, the neural mechanisms of these abnormalities are not clear but, there are plausible candidate neural circuits: altered activity of the brain midline structure (14) (agency externalization), the “where” auditory pathway (15) (spatial externalization), and the arcuate fascilcus (11) (loud experience of the inner voice).

In this present special issue, the papers expand on current knowledge, provide further insight about the mechanisms of altered experiences of the self, and suggest methodological advances in this line of research.

Rosen et al. shed some light on how an altered experience of self can come about. They have examined the interrelatedness between absorption, inner speech, self, and psychopathology and found that hallucinations and other psychotic symptoms were associated with absorption. Absorption describes a state of immersion in mental imagery and is known to be associated with vivid imagination or fantasy. The authors hypothesized that during a period of intense absorption ‘objects of attention' acquire an importance and intimacy that are normally reserved for the self and may therefore acquire a self-like quality. The findings suggest a porous boundary between self-thoughts, self-other, and the immergence of hallucinations as individuals search for meaning of their experience.

Silverstein and Lai review the nature and frequency of visual hallucinations and distortions, associated neuro-ophthalmology and neuropsychiatry, potential cortical mechanisms, associated retinal changes, and laboratory measures of visual processing. Integrating the findings across level, they suggest that to the extent that the content of visual hallucinations reflects core vulnerabilities of the self (e.g., security/danger concerns), the content of these hallucinations can be used to explore and modify dysfunctional cognitive schemata and/or to generate insight into interpersonal and existential vulnerabilities in the maintenance of a sense of self.

On a related issue, Gierch et al. describes a tool currently in the testing stage, the Strasbourg Visual Scale (SVS). SVS allows the exploration of visual hallucinations circumventing the difficulties of verbal descriptions. Simple images are displayed on a computer screen one at time with questions focusing on one physical property of the image. The successive choices made by the participants help them progressively build an image close to his/her hallucination. This tool could be particularly useful in individuals with schizophrenia who, particularly in the acute phase, have instability in their bodily self-experience, an inability to distinguish one's own self-boundary from the external world.

Storchak et al. using simultaneous functional near-infrared spectroscopy (fNIRS) and functional magnetic resonance imaging (fMRI) measurements, they examined brain activation associated with an inner speech and auditory verbal imagery in healthy subjects. Both fNIRS and fMRI exhibited congruent activations in key brain areas that have been associated with monitoring processes, which are implicated with verbal hallucinations. Importantly, the activations were dependent more on the sentence form and less on the imaging condition. More activation was noted with second person sentences than with first person sentences. The findings point to a need for accounting for the pronoun factor in inner speech imaging studies. Interestingly, hallucinations are usually reported in second and third person but not with first person (16).

López-Silva et al. address the challenges of the characterizations of the pseudohallucinations, traditionally defined based on internal experience of hallucinations. They suggested that future conceptualisations should be able to integrate the nuance and complexity of hallucinatory experiences, going beyond the rather arbitrary distinctions between inner/outer perceptual space as they are often dynamic and temporally evolving phenomena.

This issue also includes papers on topics indirectly related to the experience of self. Blom et al. examined the experiences of time distortions in Alice in Wonderland Syndrome in populations with neurological or psychotic illnesses. Forty-percentage of subjects in their sample experienced time distortion and those with psychosis had the most bizarre experiences. Furthermore, the phenomenological characteristics of tactile and somatic hallucinations in a group of Muslim patients who had severe psychotic symptoms (Lim and Blom), and the relationships between psychosis, maladaptive priors and reduced updating of these priors during perception (Benrimoh et al.).

Finally, for a progress in the understanding the altered sense of self in hallucinations, we suggest a framework for future research whereby altered self experiences are first identified through phenomenological research and the biological and psychosocial underpinnings subsequently examined.

## Author contributions

MS drafted the editorial and CH, CR, and AM reviewed and suggested changes. All authors contributed to editing and authoring the articles. All authors contributed to the article and approved the submitted version.

## Funding

This study receives funding support from VA Medical Center.

## Conflict of interest

The authors declare that the research was conducted in the absence of any commercial or financial relationships that could be construed as a potential conflict of interest.

## Publisher's note

All claims expressed in this article are solely those of the authors and do not necessarily represent those of their affiliated organizations, or those of the publisher, the editors and the reviewers. Any product that may be evaluated in this article, or claim that may be made by its manufacturer, is not guaranteed or endorsed by the publisher.

## References

[1] Parnas J Henriksen M. Disturbance of the experience of self—a phenomenologically based approach. In:Waters F Stephane M, editors. The Assessment of Psychosis: A Reference Book and Rating Scales for Research and Practice. New York, NY: Routledge (2014).

[2] Stephane M. The self, agency and spatial externalizations of inner verbal thoughts, and auditory verbal hallucinations. Front Psychiatry. (2019) 10:668. 10.3389/fpsyt.2019.00668 31607965 PMC6768100

[3] Stephane M Kuskowski M McClannahan K Surerus C Nelson K. Evaluation of speech misattribution bias in schizophrenia. Psychol Med. (2010) 40:741–8. 10.1017/S003329170999081X 19719896

[4] Laroi F de Haan S Jones S Raballo A. Auditory verbal hallucinations: dialoguing between the cognitive sciences and phenomenology. Phenomenol Cogn Sci. (2010) 9:225–40. 10.1007/s11097-010-9156-0

[5] Descartes R. Méditations métaphysiques. Paris: Flammarion (1992).

[6] James W. Principles of Psychology. New York, NY: Henry Holt and Company (1890).

[7] Damasio A. Self Comes to Mind: Constructing the Conscious Brain. New York, NY: Pantheon Books (2010).

[8] Gallagher S. Philosophical conceptions of the self: implications for cognitive science. Trends Cogn Sci. (2000) 4:14–21. 10.1016/S1364-6613(99)01417-5 10637618

[9] Stephane M Kuskowski M McClannahan K Surerus C Nelson K. Evaluation of Inner-outer space distinction and verbal hallucinations in schizophrenia. Cogn Neuropsychiatry. (2010) 15:441–50. 10.1080/13546801003619884 20349369

[10] Stephane M Thuras P Nassrallah H Georgopoulos AP. The internal structure of the phenomenology of auditory verbal hallucinations. Schizophr Res. (2003) 61:185–93. 10.1016/S0920-9964(03)00013-6 12729870

[11] Stephane M Dzemidzic M Yoon G. Altered corollary discharge in the auditory cortex could reflect louder inner voice experience in patients with verbal hallucinations, a pilot fMRI study. Schizophr Res. (2022) 243:475–80. 10.1016/j.schres.2022.02.007 35277315

[12] López-Silva P Harrow M Jobe TH Tufano M Harrow H Rosen C. ‘Are these my thoughts?': A 20-year prospective study of thought insertion, thought withdrawal, thought broadcasting, and their relationship to auditory verbal hallucinations. Schizophr Res. (2022). S0920-9964(22)00277-8. 10.1016/j.schres.2022.07.005. [Epub ahead of print]. 35945121

[13] Humpston CS Adams RA Benrimoh D Broome MR Corlett PR Gerrans P. From computation to the first-person: auditory-verbal hallucinations and delusions of thought interference in schizophrenia-spectrum psychoses. Schizophr Bull. (2019) 45(Suppl_1):S56–66. 10.1093/schbul/sby073 30715542 PMC6357975

[14] Qin P Northoff G. How is our self related to midline regions and the default-mode network? Neuroimage. (2011) 57:1221–33. 10.1016/j.neuroimage.2011.05.028 21609772

[15] Stephane M Dzemidzic M Yoon G. Keeping the inner voice inside the head, a pilot fMRI study. Brain Behav. (2021) 11:e02042. 10.1002/brb3.2042 33484101 PMC8035434

[16] Stephane M Pellizzer G Roberts S McClannahan K. Computerized binary scale of auditory speech hallucinations (cbSASH). Schizophr Res. (2006) 88:73–81. 10.1016/j.schres.2006.05.020 16901679

